# Exhausted Parents: Development and Preliminary Validation of the Parental Burnout Inventory

**DOI:** 10.3389/fpsyg.2017.00163

**Published:** 2017-02-09

**Authors:** Isabelle Roskam, Marie-Emilie Raes, Moïra Mikolajczak

**Affiliations:** Psychological Sciences Research Institute, Université Catholique de Louvain Louvain-la-Neuve, Belgium

**Keywords:** parent, burnout, exhaustion, questionnaire, test, psychometrics

## Abstract

Can parents burn out? The aim of this research was to examine the construct validity of the concept of parental burnout and to provide researchers which an instrument to measure it. We conducted two successive questionnaire-based online studies, the first with a community-sample of 379 parents using principal component analyses and the second with a community- sample of 1,723 parents using both principal component analyses and confirmatory factor analyses. We investigated whether the tridimensional structure of the burnout syndrome (i.e., exhaustion, inefficacy, and depersonalization) held in the parental context. We then examined the specificity of parental burnout vis-à-vis professional burnout assessed with the Maslach Burnout Inventory, parental stress assessed with the Parental Stress Questionnaire and depression assessed with the Beck Depression Inventory. The results support the validity of a tri-dimensional burnout syndrome including exhaustion, inefficacy and emotional distancing with, respectively, 53.96 and 55.76% variance explained in study 1 and study 2, and reliability ranging from 0.89 to 0.94. The final version of the Parental Burnout Inventory (PBI) consists of 22 items and displays strong psychometric properties (CFI = 0.95, RMSEA = 0.06). Low to moderate correlations between parental burnout and professional burnout, parental stress and depression suggests that parental burnout is not just burnout, stress or depression. The prevalence of parental burnout confirms that some parents are so exhausted that the term “burnout” is appropriate. The proportion of burnout parents lies somewhere between 2 and 12%. The results are discussed in light of their implications at the micro-, meso- and macro-levels.

## Introduction

Imagine Cecilia, a nurse who used to be very engaged in her job. She was well aware of the importance of her mission and strove to provide good emotional and medical care to her patients. She gave the best she could each and every day. For years, she coped with the heavy workload, the tiring shift work schedule and the poor rewards for her efforts. But over recent months, she has had to work even harder: one of her colleagues is on maternity leave and another one is a newcomer who has not yet mastered the tasks of the role. Her workload has increased drastically and her boss does not share the burden. She feels more and more exhausted. As she lacks the time to do her work properly, she keeps the care she provides to a strict minimum: patients' physical care. She does not have the time or energy to listen to or deal with patients' emotional difficulties. She has gradually started to consider patients as “rooms” rather than humans. As time passes, she is becoming less and less happy and people are starting to complain about her work. Most psychologists and general practitioners would detect that Cecilia is suffering from job burnout. She presents all three core symptoms of burnout: she is emotionally exhausted, she depersonalizes her patients and she is less efficient.

Now imagine Charlotte, a mother of three children who used to be there all the time for them. She was well aware of the importance of parenting and strove to provide them with optimal care and support in every way. She gave the best she could each and every day. For years, she looked after them, drove them to school and to extra-curricular activities, prepared meals, oversaw their homework, and was there for them in their happiness and sorrows. But over recent months, things have become difficult. Her eldest child has had an accident and needs physical therapy three times a week. Her youngest has entered primary school and is experiencing severe learning difficulties. Her workload as a mom has dramatically increased and her husband cannot share the burden. He comes back home late in the evening and frequently travels abroad. Charlotte feels more and more exhausted. She strives to maintain the routine: work, journeys, meals, and homework. But she does not have the time or energy to spend quality time with the children, and still less to listen to or deal with their emotional difficulties. She has become cold and irritable, and the children complain that she is not “the same as she used to be.” She feels like she is a terrible mother. Is Charlotte suffering from parental burnout?

Although the parallel between the above situations is obvious, and although many popular authors and a few scientists have already suggested the existence of parental burnout (Pelsma, 1989; Norberg, 2007, 2010; Lindström et al., 2011; Lindahl Norberg et al., 2014), the claim that Charlotte suffers from “parental burnout” can only be justified if two conditions are met. The first is that parental burnout can be precisely defined and specifically measured. And the second is that Charlotte meets the criteria and scores above the clinical cut-off level. Neither of these conditions has been met so far. Although we know, thanks to seminal work by Pelsma (1989) and by Norberg et al. (Norberg, 2007, 2010; Lindström et al., 2011; Lindahl Norberg et al., 2014), that parents can burnout because of parental issues, it is still debatable whether a specific diagnosis of “parental burnout” makes sense. Moreover, although Pelsma paved the way by suggesting that the Maslach Burnout Inventory may be a good starting point to build a measure of parental burnout, we do not yet have a fully validated instrument for this purpose.

Accepting the existence of parental burnout requires both *proximity to* and *distinctiveness from* job burnout. Conceptually speaking, the first condition to validate the existence of “parental burnout” is to show that the tridimensional structure of the burnout syndrome (i.e., exhaustion, depersonalization and inefficacy) can also be found in the case of “parental burnout” when all items refer unambiguously to the parental context. This is not self-evident (Pelsma, 1989), but is nonetheless a prerequisite to call parental burnout “burnout.” Provided that the first condition is met, the second condition is to demonstrate the distinctiveness of parental burnout from professional burnout. If this is not the case, we will have to conclude that there is a generic, context-free “burnout” phenomenon. This may also be theoretically problematic for the construct of professional burnout. The third condition is to show that parental burnout is something other than just parental stress or depression. Because burnout is situated on a continuum between stress and depression (Iacovides et al., 2003; Hakanen et al., 2008), a partial overlap is expected, but it should be moderate. If all these three conditions are met, the last condition is to show that “parental burnout,” as its name suggests, is not limited to mothers; otherwise it should be named “maternal burnout” or “mothering burnout.”

In light of the foregoing, the aim of this research was to examine the construct validity of the concept of parental burnout and, should it be deemed valid, to provide researchers with an instrument to measure it. We accumulated theoretical and empirical evidence in favor of the existence of parental burnout. Before presenting the empirical research and results, we shall briefly introduce the notion of job burnout. In addition to being helpful for readers who are not familiar with job burnout research, this will allow them to realize the parallels that can be drawn between job burnout and parental burnout.

## The notion of burnout

As will be the case for parental burnout, the use of the term “burnout” and lay descriptions of the syndrome appeared *before* the scientific community took it up as an object of study. As pointed out by Maslach and Leiter, “the importance of [job] burnout as a social problem was identified by both practitioners and social commentators long before it became a focus of systematic study by researchers” (Maslach et al., 2001, p. 398). Another salient parallel with parental burnout is that the increasing prevalence of job burnout in the workplace since the 70s was preceded by a number of socio-cultural changes that resulted in fundamental transformations in the workplace and the nature of jobs (Maslach and Leiter, 1997; Schaufeli et al., 2009). As we will explain in the section on parental burnout, these changes were mirrored in the parental context a few decades later, and it is no coincidence that parental burnout emerged in the 2000s.

Although cases of burnout were described and analyzed by Freudenberger (1974), it is to Maslach (1976) that we owe the conceptualization of burnout that still prevails. After 10 years of qualitative and quantitative research, she proposed a conceptualization of job burnout as a psychological syndrome encompassing three key dimensions: overwhelming exhaustion, a depersonalization of the beneficiaries of one's work, and a sense of ineffectiveness and lack of accomplishment (Maslach and Jackson, 1981; Maslach, 1993). The exhaustion component represents the core dimension of burnout. It refers to feelings of being overextended and depleted of one's emotional and physical resources. The depersonalization component refers to a negative, callous, or excessively detached response to various aspects of the job, including the beneficiaries of one's work. The component of reduced efficacy or accomplishment refers to feelings of incompetence and a lack of achievement and productivity at work (Maslach et al., 2001).

## From job burnout to parental burnout

The first traceable account of parental burnout dates from 1983 in the testimony of Edith Lanstrom, a Christian mother, in her book *Christian parent burnout*. That same year, a university professor specializing in educational leadership published a book together with a leadership consultant with whom he was conducting workshops on job burnout. The book, entitled *Parent burnout*, argued that parenting can lead to exhaustion to such a degree that it could be called burnout (Procaccini and Kiefaver, 1983). The authors concurred with Freudenberger's individualistic vision of the etiology of burnout: parents who burn out are those who looked forward to parenthood the most, who give it their all, in a word: overcommitted, overzealous parents. A few years later, Pelsma examined the validity of the Maslach Burnout Inventory for assessing parental burnout in a 100 non-working mothers of young children. He found support for two of the three dimensions (emotional exhaustion and lack of personal accomplishment) but not for the third one (depersonalization). The paper was published in 1989 but remained largely unnoticed. Apart from these three accounts, there was no other publication on the subject until the 2000s. At that time, the topic of parental burnout did not seem to resonate in the target audience.

While the job burnout wave hit the USA in the 70s, the parental burnout wave hit Europe in the 2000s. Interestingly, the socio-cultural changes that occurred in the parenting domain in the 90s in Europe seemed to mirror the changes in the human services work domain in the USA in the 60s (see Schaufeli et al., 2009 for a brief account of the latter). The changes in the human services work comprised five elements: firstly, the definition by the state authorities of a noble target (the *War on poverty* decreed by President Johnson in 1964) that was impossible to reach in practice (there will always be poverty), leading to frustrated idealism. Secondly, and relatedly, the increased state incursion in job descriptions: jobs that were originally a “calling” began to be highly formalized. Thirdly, the weakened professional authority of human services workers (doctors, nurses, teachers, police officers, etc.) gradually resulted in less respect from beneficiaries (see e.g., Pescosolido et al., 2001). Fourthly, the expectations of empowered beneficiaries regarding care, service, empathy and compassion rose drastically (see e.g., Lateef, 2011). Fifthly, the economic crises of the 1970s meant that people were trying to achieve these impossible goals with fewer resources (in terms of equipment, personnel, etc.).

The very same factors were at play in the parenting domain in the decade preceding the emergence of the term “parenting burnout” in the media in Europe. Firstly, the definition by the state authorities of a noble target (*Positive parenting*, consisting of non-violent, warm, supportive and sensitive parenting valuing children as people in their own rights, as decreed by the Council of Europe in 2007, Daly, 2007), that was impossible to reach in practice (it is impossible to apply all these principles at all times), leading to frustrated idealism. Secondly, and relatedly, the increased state incursion in parenthood: while parenting was formerly done with love and common sense, the exercise of parental authority became increasingly a subject of legislation (e.g., spanking legislation, duRivage et al., 2015). Thirdly, the weakened authority of parents (due to a focus on children's rights and parents' duties) resulted in less respect from children (Richards, 2010). Fourthly, the expectations of empowered children in terms of attention, education, possessions and opportunities rose drastically (Daly, 2007). Fifthly, the drastic increase in woman's work (+75% between 1980 and 2010; ec.europa.eu/eurostat/statistics) meant that parents were trying to achieve these impossible goals with less time.

In summary, the increased pressure on parents, combined with the lack of time due to the drastic decrease in stay-at-home mothers and the lower respect and/or appreciation from children, has made parenting increasingly challenging. It is therefore no coincidence that parental burnout emerged in Europe in the 2000s. As was the case with job burnout, practitioners and journalists identified the importance of parental burnout before it became a focus of systematic study by researchers. It has not yet become a central focus for researchers, but research has started to emerge. In 2007, Norberg assessed burnout using the Shirom–Melamed Burnout Questionnaire (SMBQ) among 24 mothers and 20 fathers of childhood brain tumor survivors, and compared their scores to those of parents of children with no history of chronic or serious diseases (Norberg, 2007). Mothers' burnout scores were significantly higher compared to those of reference mothers. There was a non-significant tendency in the same direction for fathers. In 2010, her team replicated these results on a sample of 252 parents of children with Type 1 Diabetes Mellitus and 38 parents of children with Inflammatory Bowel Diseases, whose scores on the SMBQ were compared to that of 124 randomly selected parents of healthy children. Again, mothers' burnout scores were significantly higher compared with reference mothers; for fathers, there was only a non-significant tendency in the same direction (Lindström et al., 2011). In a follow-up study carried out 7 months later on the parents of childhood brain tumor survivors, Norberg (2010) showed that burnout was predicted by parents' perception of the influence of the disease on their everyday life. These results were refined in another publication about the sample of parents of children with Type 1 Diabetes Mellitus, in which the team showed that the predictors of parental burnout were not sociodemographic or medical factors but low social support, lack of leisure time, financial concerns and a perception that the child's disease affected everyday life. Additional predictors in mothers were low self-esteem and high need for control (Lindström et al., 2011). In their last study (Lindahl Norberg et al., 2014), they compared the SMBQ scores of 159 mothers and 123 fathers of children who had undergone and survived pediatric hematopoietic stem cell transplantation (HSCT). Burnout occurred more often among fathers of children who had undergone transplantation within the last 5 years than among fathers of children with no history of serious disease. Among both mothers and fathers, burnout was predicted by the child's number and severity of health impairments during these 5 years.

Although these studies strongly suggest the existence of parental burnout, its existence cannot be formally ascertained yet, because the questionnaire used to measure burnout (MSBQ) contains 10 context-free items (e.g., I feel fed up; I feel physically drained; my thinking process is slow) and four job-related items (e.g., I feel I am not capable of investing emotionally in coworkers and customers). Therefore, these studies do not provide information about the validity and specificity of *parental* burnout or on the prevalence of this specific form of burnout in the general population. Note that although this is a weakness from the standpoint of parental burnout research, it is not a weakness of these studies *per se*, as they did not intend to document the existence of parental burnout; rather, they sought to show how much and for how long parents could be affected by children's severe health issues, even after the child's recovery.

## Aim of the current research

The aim of this research was to examine the construct validity of the concept of parental burnout and, should it be deemed valid, to provide researchers with an instrument to measure it, as well as norms to interpret scores in an exploratory way. To this end, we first investigated whether the tridimensional structure of the burnout syndrome (i.e., exhaustion, depersonalization and inefficacy) could also be found in the case of “parental burnout.” We then examined the specificity of parental burnout vis-à-vis professional burnout, parental stress and depression. Next, we checked if parental burnout concerned both genders or if it should be renamed “*Mothering burnout*.” Finally, we determined cut-off scores and examined the prevalence of parental burnout in the general population.

## General method

### Overview

The two studies reported here are part of the *BParent* research program conducted at the Université catholique de Louvain in Belgium which received the approval of the Ethics Committee of the Psychological Sciences Research Institute. *BParent* is a recent research program focusing on the nature, causes and consequences of parental burnout. Participants in the two studies were informed about this research program through social networks, websites, schools, pediatricians or by word of mouth. Inclusion criteria was to be parent and to have at least one child still leaving at home. In order to avoid (self-)selection bias, participants were not informed that the study was about parental burnout. Study 1 was presented as a study about work-family balance (this ensured that all participants were working parents, which was important as we aimed to examine the specificity of parental burnout vis-à-vis professional burnout). Study 2 was presented as a study about “being a parent in the twenty-first century” (we aimed to recruit a wider sample, including unemployed parents). Parents could participate in the studies only if they had (at least) one child still living at home. Participants were invited to complete an online questionnaire after giving informed consent. The informed consent signed by the participants allowed them to withdraw at any stage without having to give any justification. They were also assured that data would remain anonymous. Participants who completed the questionnaire had the opportunity to enter a lottery with a 1/1000 chance of winning €200. Participants who wished to participate in the lottery had to provide their email address, but the latter was disconnected from their questionnaire.

A potential measure of parental burnout was assessed in both studies. A preliminary version of the Parental Burnout Inventory (PBI) was created and used in Study 1. This version was an adaptation of the Maslach Burnout Inventory (MBI; Maslach and Jackson, 1981), in which each of the 22 items of the MBI was adapted to fit the parenting context. For example, “I feel emotionally drained from my work” was changed into “I feel emotionally drained from my parental role.” In Study 2, refinement of the PBI led us to reconsider items from the depersonalization factor. Eleven new items presented in the Table S2 were proposed relating to the concept of emotional distancing, which appeared to be more suited to parental context than depersonalization. The idea of replacing depersonalization with emotional distancing emerged from the discussions of two 1-h focus groups (*n*1 = 12, *n*2 = 8) that we set up with colleagues to discuss the results of Study 1 (and in particular the poor validity of the depersonalization component in the parental context). Four questions had been prepared by the facilitators: (1) Do you think that depersonalization exists in parental burnout? (2) If yes, what are the core characteristics of depersonalization in parental burnout? (3) If not, is there another specific mechanism in parental burnout (try to name it)? (4) What are its characteristics? Exactly the same idea (i.e., that depersonalization takes the form of emotional distancing in the parental context) emerged from the two focus groups. The 11 “emotional distancing” items were then created and refined together with 8 colleague-parents who participated in these groups. Parental burnout was therefore reassessed in Study 2 using a set of 28 items, leading to a final 22-item version assessing emotional exhaustion (8 items), emotional distancing (8 items) and personal accomplishment (6 items). In both studies, PBI items were rated on the same 7-point Likert scale as in the original MBI: never (0), a few times a year or less (1), once a month or less (2), a few times a month (3), once a week (4), a few times a week (5), every day (6). Factor and global scores were obtained by summing the appropriate item scores, with higher scores indicating greater burnout; the items of the personal accomplishment factor were therefore reverse-scored.

### Data analyses

The questionnaire was completed online with the forced choice option, ensuring a dataset without missing data. The validation of the PBI was conducted according to the standards and guidelines of the American Psychological Association (AERA et al., 2014). In the current paper, we provide evidence to support the internal structure of the PBI and its relations to other variables.

With regard to the internal structure of the PBI, principal components analyses (PCAs), parallel analyses (Horn, 1965), reliability estimates and assessment of normality were performed in the two studies with Factor 10.2 software (Lorenzo-Seva and Ferrandon, 2013) in order to test if the three-factor structure of the MBI replicated in a parenting context. Only factor loadings higher than 0.30 were interpreted. Parallel analyses based on 500 random permutations of the original data were used in order to determine how many components to extract. These analyses were based on a comparison between eigenvalues from a factor analysis of the actual data and eigenvalues from a factor analysis of a random dataset. The number of components to be retained was based on the number of actual data eigenvalues higher than the upper 95% confidence limit of random data eigenvalues (Horn, 1965). Reliability was estimated with Cronbach's alpha coefficients (α). Assessment of normality was based on skewness and kurtosis values. Values of asymmetry and kurtosis between −2 and +2 were considered sufficient to prove normal univariate distribution (George and Mallery, 2010). Confirmatory factor analyses (CFAs) in Study 2 were computed using SEM software AMOS 18.0 (Arbuckle, 1995, 2007). The measurement model included the three latent variables representing the concepts of emotional exhaustion, emotional distancing and personal accomplishment, and their indicators consisting of 8 items for emotional exhaustion, 8 for emotional distancing and 6 for personal accomplishment. Analyses were conducted based on the covariance matrix and using maximum likelihood estimation. Several goodness-of-fit indices were used to determine the acceptability of the models: χ^2^, the comparative fit index (CFI) (Marsh et al., 1988) and the root mean-square error of approximation (RMSEA) (Byrne, 2001). Chi-square compares the observed variance-covariance matrix with the predicted variance covariance matrix. It theoretically ranges from 0 (perfect fit) to ∞ (poor fit). It is considered as satisfactory when it is non-significant (Byrne, 2001). χ^2^/df is considered as satisfactory when it is <2.5 in medium-sized samples (100 < N < 200) (Hu and Bentler, 1999; Byrne, 2001). Note that the use of chi-square in a large sample may be inadequate because excessive test power (because of the large N) may prompt the rejection of acceptable models (Hayduk, 1996). For CFI, values close to 0.90 or greater are acceptable, while values higher than 0.95 indicate a good fit to the data. RMSEA should preferably be less than or equal to 0.06, but values under 0.08 are acceptable (Hu and Bentler, 1999).

With regard to the relations between the PBI and other variables, the specificity of parental burnout vis-à-vis close constructs was investigated by examining its correlations with professional burnout and depression in Study 1, and parental stress in Study 2. Also in Study 1, in order to test if the concept of burnout was context-specific, both professional and parental burnout items were subjected to a PCA, on the expectation that six factors would be extracted with professional and parental items loading on different dimensions. The relationship with sociodemographic variables and criterion variables was also examined but will be described elsewhere (in a paper on the antecedents of parental burnout).

## Study 1

### Sample

Data were collected from a sample of 379 parents. The characteristics of the sample are presented in Table 1.

**Table 1:** **Characteristics of the sample in study 1 and study 2**.

	**Study 1 (*n* = 379)**	**Study 2 (*n* = 1,723)**
Number of women	314 (83%)	1499 (87%)
Parent age	M = 39.92 (*SD* = 7.55) [22–61]	M = 39.50 (*SD* = 8.26) [22–75]
Children age	[0–38]	[0–38]
Number of siblings	M = 2.23 (*SD* = 0.98) [1–7]	M = 2.30 (*SD* = 1.05) [1–7]
Number of children with chronic illness or disability	52 (13.7%)	194 (11.3%)
**MARITAL STATUS (N)**		
Living with a partner	328 (86.5%)	1453 (84.3%)
Single parents	51 (13.5%)	270 (15.7%)
**EDUCATIONAL LEVEL (N)**		
12 years (compulsory education)	62 (16.3%)	262 (15.2%)
15 Years (undergraduates)	149 (39.1%)	602 (35.2%)
>15 years	168 (44.6%)	855 (49.6%)
**NET MONTHLY INCOMES (N)**		
<2500€	68 (17.7%)	388 (22.4%)
2500–4000€	162 (42.8%)	735 (42.7%)
4000–5500€	94 (24.5%)	421 (24.4%)
>5500€	55 (15%)	179 (10.5%)

### Measures

#### Socio-demographics

Socio-demographics participants were asked about their age, gender, number of children, nationality, marital status (single, cohabitant, married, divorced, widowed), type of family (single parent, living with the father/mother of the children, step-family), level of education, and net monthly household income.

#### Professional burnout

Professional burnout was assessed with the Maslach Burnout Inventory (MBI) (Maslach and Jackson, 1981). The MBI is a widely used 22-item questionnaire encompassing three factors, i.e., emotional exhaustion (9 items), depersonalization (5 items) and personal accomplishment (8 items). Items are in the form of “I feel emotionally drained from my work.” The instruction is as follows: “Please read each statement carefully and decide if you ever feel this way about your job.” Likert-type scales are in the form of “How often,” with a 7-point scale of frequency, i.e., never (0), a few times a year or less (1), once a month or less (2), a few times a month (3), once a week (4), a few times a week (5), every day (6).The global score is computed after reversing the items of the personal accomplishment factor, so that higher scores indicate greater burnout. The Cronbach alphas reported in the MBI Manual are 0.90 for emotional exhaustion, 0.79 for Depersonalization, and 0.71 for Personal accomplishment (Maslach et al., 2010). The Cronbach alphas found in the current sample were 0.90, 0.69, and 0.75, respectively.

#### Parental burnout

Parental burnout was measured using a version of the MBI adapted to the parental context, as previously explained in the Overview of the General Method.

#### Depression

Depression was assessed with the short form of the Beck Depression Inventory (BDI). The BDI is a 13-item, self-report questionnaire measuring symptoms of depression (Beck et al., 1961). Items are scored on a scale from 0 to 3, e.g., 0: “I do not feel sad,” 1: “I feel sad,” 2: “I am sad all the time and I can't snap out of it,” 3: “I am so sad and unhappy that I can't stand it.” The depression score is obtained by summing the 13 item scores. Internal consistency for the BDI ranges from 0.73 to 0.92 with a mean of 0.86 (Beck et al., 1988). The Cronbach alpha found in the current sample was 0.86.

### Results

#### Factor structure and reliability

Parallel analyses conducted on the 22 items supported a two-factor structure when the means of random eigenvalues were considered and a three-factor structure when 95th percentile random data eigenvalues were considered. The first five eigenvalues from the actual data were 7.65, 2.87, 1.32, 1.12, and 1.01; the corresponding first five means of random eigenvalues were 1.45, 1.38, 1.32, 1.26, and 1.22; the corresponding 95th percentile random data eigenvalues were 1.53, 1.44, 1.36, 1.30, and 1.26.

Since the items were adapted from the three factors of the MBI, three components were retained for the PCA of the 22 items. The three components explained 53.96% of the variance. The loading parameter estimates for the three- components solution as well as reliability estimates are presented in the Table S1. All the items of the emotional exhaustion and personal accomplishment scales had their primary loading on the expected component with no cross-loading. However, for depersonalization, only two items had their primary loading on the expected component with no cross-loading (DP2 and DP3). Two others had their primary loading on emotional exhaustion component (DP1 and DP5), and the last did not load on any of the three components (DP4). Fixing this problem was therefore the main focus of Study 2. Skewness and kurtosis indicated that three of the 22 items displayed deviations from normality. These three items were the three depersonalization items that did not load on the expected component or did not load on any of the three components.

In order to investigate the relations between PBI and other variables, scores were computed for the emotional exhaustion and decreased personal accomplishment subscales, but not for depersonalization, the validity of which was not supported by the PCA. Scores were obtained by summing the item scores of the two subscales (reverse-scored for personal accomplishment); the higher the score, the greater the burnout. Descriptive statistics of mean scores are presented in Table 2. Due to deviations from normality, non-parametric correlations with both professional burnout and depression were computed for the two validated subscales only.

**Table 2:** **Descriptive statistics of mean scores of the PBI subscales in study 1 and study 2**.

	**Study 1 (*****N*** = **379)**			**Study 2 (*****N*** = **1,723)**		
	***M***	***SD***	**Range**	***M***	***SD***	**Range**
PBI_Emotional exhaustion	8.44	10.16	0–44	15.77	11.62	0–48
PBI_Personal accomplishment	9.99	6.93	0–41	7.00	5.20	0–48
PBI_Emotional distancing	–	–	–	8.28	7.61	0–33
PBI_Total score	–	–	–	31.05	19.38	0–108

#### Relationships with other variables

Correlation coefficients between parental burnout, professional burnout and depression are presented in Table 3. Low to moderate coefficients suggest that as expected, significant relations exist between parental burnout, professional burnout and depression, but also that there is no complete overlap between the concepts under consideration, i.e., parental burnout is not just burnout and it is not just depression.

**Table 3:** **Correlations between parental burnout and professional burnout, depression, and parental stress**.

	**Parental burnout**			
	**Emotional exhaustion**	**Decreased personal accomplishment**	**Emotional distancing**	**Total score**
**PROFESSIONAL BURNOUT**				
Emotional exhaustion	0.42^***^	0.20^***^	–	–
Decreased personal accomplishment	−0.01	0.20^***^	–	–
Depersonalization	0.17^***^	0.16^***^	–	–
Depression	0.48^***^	0.41^***^	–	–
**PARENTAL STRESS**				
Parent-child relationship problems	0.53^***^	0.45^***^	0.45^***^	0.62^***^
Parenting problems	0.37^***^	0.53^***^	0.40^***^	0.53^***^
Parental role restriction	0.54^***^	0.02	0.24^***^	0.41^***^

*** *p < 0.001*.

The PCA exploring components of both the 22 professional and the 22 parental burnout items showed that, as expected, the concept of burnout was context-specific, with professional and parental items loading on separate components, i.e., three for professional and three others for parental burnout. The only exception was for “I feel I look after my children impersonally, as if they are objects,” which loaded on professional depersonalization. Parallel analyses conducted on the 44 items suggested a five-factor structure when both the means of random eigenvalues and the 95th percentile random data eigenvalues were considered. The first six eigenvalues from the actual data were 10.14, 4.62, 4.18, 1.98, 1.74, and 1.26; the corresponding first six means of random eigenvalues were 1.75, 1.66, 1.60, 1.55, 1.50, and 1.46; the corresponding 95th percentile random data eigenvalues were 1.83, 1.72, 1.65, 1.59, 1.54, and 1.50. This was however due to the sixth component of parental depersonalization which was found to be problematic.

## Study 2

### Sample

Data were collected from a sample of 1,723 parents. The characteristics of the sample are presented in Table 1.

### Measures

#### Socio-demographics

Socio-demographics participants were asked about their age, gender, number of children, nationality, marital status (single, cohabitant, married, divorced, widowed), type of family (single parent, living with the father/mother of the children, step-family), level of education, and net monthly household income.

#### Parental burnout

Parental burnout was measured using a revised 28-item version of the questionnaire used in Study 1, as previously mentioned in the Overview of the General Method. Following the results of Study 1, the 9 items of the exhaustion scale and the 8 items of the personal accomplishment scale were kept. All items of the depersonalization scale were dropped and replaced by 11 new items created to reflect emotional distancing instead of depersonalization.

#### Parental stress

Parental stress was assessed with the Parental Stress Questionnaire (PSQ) (Vermulst et al., 2011). In its original form, this 34-item questionnaire includes five factors, i.e., parent-child relationship problems, parenting problems, depressive mood, parental role restriction, and physical health problems. In order to limit the number of items in the online survey and retain only items specific to *parental* stress, only the scales “parent-child relationship problems,” “parenting problems” and “parental role restriction” were included. Items (e.g., “I feel happy with my child” or “Raising my child leaves me with too little personal time”) were rated on a four-point Likert-type scale from “not true” to “very true.” Scores were obtained by averaging the item scores in each of the subscales, with higher scores meaning higher parental stress. Cronbach alphas reported for the original version of the PSQ scales ranged from 0.74 and 0.87 (Vermulst et al., 2015). Cronbach alphas found in the current sample were 0.80, 0.72, and 0.86 for the three subscales, respectively.

### Data analyses

For factor analysis purposes, the sample was split into two subsamples of 862 and 861 participants, respectively. The 1,723 subjects were randomly assigned to one of the two subsamples. The comparability of the two subsamples was checked with crosstabs and χ^2^ analyses for categorical variables (e.g., parent gender) and with oneway ANOVAs for continuous variables (e.g., parent age). They were found to be strictly similar with regard to socio-demographic characteristics. PCA was computed on the first subsample and CFA was performed on the second subsample. Analyses testing the relations between parental burnout and other variables as well as examining the prevalence of burnout were conducted on the entire sample (*N* = 1,723).In the absence of a clinical sample of exhausted parents (Schaufeli et al., 2001), prevalence was estimated three times with three concurrent methods for comparison purposes. First it was estimated based on the professional burnout cutoff as recommended by Maslach et al. (2010). According to the cutoffs provided for each MBI subscale by Maslach et al. (2010, Appendix E, p. 48), people belong to the “low burnout” category if their global MBI score is less than 30, to the “average burnout” category if their MBI score is between 31 and 54, and to the “high burnout” category if their global MBI score is greater than 55. Second, it was estimated using a theoretical approach based on the response scale. Parental burnout was rated with a 7-point, Likert-type scale ranging from never (0) to every day (6). We theoretically considered that parents had a high level of burnout if they scored over 88, i.e., if they experienced each symptom/item at least once a week. Third, prevalence was estimated with a statistical cutoff usually used in clinical setting corresponding to 1.5 standard deviation over the mean of the sample (*N* = 1,723). These estimations were also performed separately for mothers and fathers in order to ensure that parental burnout concerned fathers as well as mothers.

### Results

#### Factor structure and reliability

Parallel analyses conducted on the 28 items suggested five-factor structure when the means of random eigenvalues were considered and a three-factor structure when 95th percentile random data eigenvalues were considered. The first seven eigenvalues from the actual data were 9.14, 4.34, 2.12, 1.47, 1.25, 1.11, and 1.08; the corresponding first seven means of random eigenvalues were 1.34, 1.29, 1.26, 1.22, 1.20, 1.17, and 1.14; the corresponding 95th percentile random data eigenvalues were 1.38, 1.33, 1.28, 1.25, 1.22, 1.19, and 1.17.

The three-factor structure which was estimated for comparative purposes explained 55.76% of the variance. The loading parameter estimates for the three-factor solution as well as the reliability estimates are presented in Table S2. All the items of the emotional exhaustion scale had their primary loading on the expected component, except for one item loading on personal accomplishment. This item was dropped. All the items of the personal accomplishment scale had their primary loading on the expected component, with only one cross-loading for one personal accomplishment item. However, one item was dropped because it was not specifically related to parenting (Pa7_ *I feel full of energy*). The other one was dropped because its French wording, i.e., *vivifié(e)*, was found to be difficult to understand by low-educated participants (PA8_ *I feel refreshed when I have been close to my children*). For emotional distancing, over the 11 new items, eight had their primary loading on the expected component with only one cross-loading. They were kept for the final version.

Skewness and kurtosis indicated that seven of the 22 final items displayed deviations from normality. Four of these items were from the emotional distancing subscale and the three others from the personal accomplishment one. Conceptually, these deviations from normality make sense: burnout is not expected to be normally distributed in the population. Like most mental health indicators, burnout is expected to present an asymmetric distribution (i.e., to be positively skewed, like most psychological disorders). However, as normality is a critical assumption underlying the maximum likelihood procedure used for CFA, log transformations of these items were computed and ensured a normal distribution. Then CFA was performed twice, once including transformed items and the other including original items. Estimates and model fit indices were strictly similar. Therefore, only the results obtained from the analyses computed on original variables are presented.

With regard to fit indices, χ^2^ was significant, $\chi_{\left(180\right)}^{2}$ = 704.12, *p* < 0.001, suggesting that a significant proportion of the variance was unexplained by the model. However, this should not necessarily lead to rejection of the model because of inadequate use of χ^2^ in a large sample. Other fit measures demonstrated a good fit to the data (CFI = 0.95, RMSEA = 0.06) with all estimated factor loadings being significant at *p* < 0.001. The completely standardized factor loadings ranged between 0.41 and 0.90. They are displayed in Table 4. Correlations between the three factors were 0.48, 0.40, and 0.67. These results provide strong support to the validity, i.e., internal structure, of the PBI.

**Table 4:** Standardized regression weights from CFA and reliability estimates for the final 22-item version^1^ in study 2.

	**ED**	**EE**	**PA**
ED1	0.587		
ED2	0.461		
ED3	0.533		
ED4	0.749		
ED5	0.671		
ED6	0.558		
ED7	0.757		
ED8	0.489		
EE1		0.815	
EE2		0.721	
EE3		0.741	
EE4		0.850	
EE5		0.767	
EE6		0.837	
EE7		0.698	
EE8		0.903	
PA1			0.414
PA2			0.434
PA3			0.587
PA4			0.879
PA5			0.722
PA6			0.726
α	0.88	0.95	0.87

*PA, Personal Accomplishment; EE, Emotional Exhaustion; ED, Emotional Distancing*.

1 *Items EE1 to EE8 and PA1 to PA6 Copyright © 1981 Christina Maslach & Susan E. Jackson. All rights reserved in all media. Published by Mind Garden, Inc., www.mindgarden.com. Altered with permission of the publisher*.

#### Relationships with other variables

In order to investigate the relations between PBI and other variables, scores were computed for the three validated PBI factors. These were obtained by summing the item scores in each of the three subscales (items of the personal accomplishment scale were reverse-scored); the higher the scores, the higher the burnout. A global score was also computed, which was found to be highly reliable (α = 0.91). Descriptive statistics of the mean scores are presented in Table 2.

Non-parametric correlations (due to deviations from normality) between the PBI and parental stress subscales are presented in Table 3. Moderate coefficients suggest that, as expected, significant relations exist between parental burnout and parental stress, but also that there is not a complete overlap between the two instruments and concepts they measure. In other words, parental burnout is not just parenting stress.

#### Prevalence of burnout

Finally, prevalence was estimated in the sample of 1,723 participants. According to the cutoff points provided for job burnout in the MBI manual (Maslach et al., 2010), 56.2% of the parents (55.3% of the mothers, 62.5% of the fathers) belonged to the “low burnout” category, 31.1% of the parents (31.8% of the mothers, 25.9% of the fathers) to the “average burnout” category and 12.7% of the parents (12.9% of the mothers, 11.6% of the fathers) to the “high burnout” category. According to the theoretical approach based on the response scale, 1.3% of the parents (1.3% of the mothers, 1.3% of the fathers) would be considered to be experiencing burnout (i.e., experiencing each symptom/item at least once a week = score over 88). Finally, according to a statistical cutoff corresponding to 1.5 standard deviation over the mean of the current sample, 8.8% of the parents (8.8% of the mothers, 8.5% of the fathers) would be considered to be experiencing burnout.

## Discussion

Important sociological changes in recent decades have increased pressure on parents to bring up healthy, secure and successful children who will become well-rounded and engaged citizens. Combined with a drastic decrease in stay-at-home mothers, these changes have made parenting both increasingly demanding and increasingly difficult. It is in this context that the concept of parental burnout started to emerge. The aim of the current research was to examine the construct validity of the concept of parental burnout and to provide researchers with an instrument to measure it. Throughout the two studies, we accumulated evidence in favor of the existence of parental burnout.

First, our study supported the three-dimensional structure of burnout in the parental context. However, a crucial step was overcome from Study 1 to Study 2 by replacing depersonalization with emotional distancing. As in Pelsma's work (1989), depersonalization items adapted to parental context from the professional burnout inventory did not work well in Study 1. Actually, it is not surprising that depersonalization was found to be unsuitable in the parental context. Although highly exhausted employees may consider their clients or patients as numbers, highly exhausted parents cannot “dehumanize” their children. Even when they are at the end of their rope, parents who do not suffer from psychosis or antisocial disorders cannot consider the flesh of their flesh as objects. What they can do, however, is distance themselves from the source of exhaustion. In our clinical and research experience with parents of children with externalized disorders, i.e., conduct disorder or antisocial behavior, we have observed that exhausted parents disengage emotionally rather than physically, i.e., they provide practical care such as feeding or sleeping but became less emotionally involved, sensitive and responsive to their offspring. This view was empirically supported by both exploratory and confirmatory factor analyses in Study 2. Thus, as in the professional context, parental burnout takes the form of a tridimensional syndrome encompassing emotional exhaustion, emotional distancing and (low) personal accomplishment. The final version of the Parental Burnout Inventory (PBI) consists of 22 items displaying strong psychometric properties with good fit to the data and high reliability estimates.

Second, our study suggests that parental burnout is, like job burnout, a specific syndrome. The overlap with professional burnout and depression was examined in Study 1, and with parental stress in Study 2. For professional burnout, coefficients ranged from −0.01 to 0.42. They were 0.41 and 0.48 for depression and ranged from 0.02 to 0.62 for parental stress subscales. These low to moderate correlations suggest that, as expected, significant relations exist between parental burnout, professional burnout, parental stress and depression, but also that parental burnout is not just burnout, stress or depression. The independence between professional and parental burnout was also supported by the PCA including both MBI and PBI items, meaning that being exhausted at work does not imply being exhausted at home. For many workers incurring burnout, family life may be seen as a safe haven and for many parents incurring burnout, work seems to be a safe place. This study therefore confirms that burnout is a context-specific rather than a context-free syndrome. Second, our study supports that the burnout syndrome in the parental context is not exclusive to mothers. Although the vast majority of our participants were women—which suggests that women may still be more involved in parenting than fathers—the study confirms that burnout concerns fathers as well. Fathers who put an effort into their fathering (i.e., they were interested in their work-family balance—Study 1—and in being a parent—Study 2) had the same probability of burnout as mothers. Hence, irrespective of the cutoff points used, the prevalence of parental burnout was the same among mothers and fathers. This suggests that the name “parental burnout” is more appropriate than “maternal burnout,” especially as fathers are becoming increasingly involved in parenting. The prevalence of parental burnout confirms Procaccini and Kiefaber's intuition that some parents are so exhausted that the term “burnout” is appropriate (Procaccini and Kiefaver, 1983). The proportion of burnout parents lies in the current study somewhere between 2 and 12% (depending on the cut-off points applied).

## Limitations and recommendations for future research

While innovative and promising, the current study is by no mean definitive. Several limitations have to be mentioned that lead to recommendations for future research.

First, Pelsma (1989) had suggested that the MBI may be a good starting point to build a measure of parental burnout. And we documented how fundamental transformations in the workplace mirrored in the parental context a few decades later. However, there may be some disadvantages of starting from an existent scale to make it stick to another concept. Another way to proceed would have been to start from parental experience as did Maslach and Freudenberger for the MBI. In this respect, the construct validity of the PBI would gain from a mix-method approach including qualitative data collection. The percentage of variance explained of 55.76% reported in study 2 may be improved by a refinement of the construct validity of the PBI through a mix-method approach.

Second, our results are compatible with the view of Hakanen et al. (2008) and Iacovides et al. (2003) that (parental) burnout may be situated on a continuum between stress and depression. But the cross-sectional design of the two studies does not permit to confirm this view. On the one hand, correlations found with parental stress are coherent with the common view that burnout occurs when high stress has been experienced for too long. On the other hand, correlations found with depression could mean that experiencing burnout may lead to abundant and long-term stress hormone secretion resulting in a depressive syndrome. Longitudinal studies of (sub)clinical samples investigating the developmental course of parental stress turning into parental burnout and depression are nevertheless needed to confirm this.

Third, none of the three methods employed in this study to estimate prevalence is fully satisfying. The first method, based on Maslach's cutoffs, implies that the professional and parental contexts share the same exhaustion boundaries. The limited overlap between parental and professional burnout suggests that this is not the case. Moreover, people can probably “bear” more in the parental context before feeling exhausted than in the professional context. The second method, based on response-scales, relies on our clinical experience from parental counseling that exhausted parents experience burnout symptoms/items at least once a week. However, social desirability may lead parents to under-report the frequency with which they experience the various symptoms/items in the questionnaire, which would make the cut-off point of 88 irrelevant. Finally, the third method, based on a statistical cutoff, is not entirely satisfying either. Although it is mainly used in psychometric approaches to IQ tests, for example, it is far less relevant in case of non-normally distributed data. Studies on incoming patients consulting for parental exhaustion are therefore urgently needed to establish clinically-relevant cutoffs, i.e., associated with actual differences in functioning. However, since parental burnout is a very recent concern in the European countries (see the discussion of socio-cultural changes in the introduction section), parental burnout is just emerging as a reason for referral. Several months (or years in the US) would probably be needed to gather participants referred for severe parental exhaustion. Future studies are needed to refine these estimations based on clinically-derived cutoffs.

Fourth, percentage of mothers and fathers belonging to high burnout category irrespective of the cutoffs used, was found to be similar. However, we did not test the measurement invariance across genders in order to ensure that the same construct was assessed in mothers and fathers. We could not test as we had only 224 men in the study. Future studies should replicate our preliminary results and test measurement invariance across gender. They should also take into account possible confounding variables. Indeed, statistical models as one-way MANCOVA may be used to assess differences among the frequency to which parental burnout are displayed by mothers and fathers when other variables are controlled for. In particular, age or personality traits as neuroticism should be taken into account. The same models may be implied to parents having chronically ill or disabled child and parents having healthy children, as well as to parents having infants or preschoolers and parents with older children. The differences among the frequency to which parental burnout is displayed in subgroups has to be investigated to document that parental burnout is a problem of general interest and not a problem specific to some subgroups of parents only.

Finally, considering the consequences parental burnout may have for the individual, the couple, and the child(ren), studies addressing this syndrome are urgently needed. As well as the consequences, the causes also need to be examined, in order to understand the developmental processes that underlie parental burnout and define intervention strategies. Although the causes and consequences of parental burnout lay outside the scope of the current study, we will now put forward a few hypotheses about causes and consequences at the micro-, meso- and macro-levels that should be tested in future research.

As a main causal mechanism, any sociodemographic, family or personal characteristics that increase the imbalance of demands over resources should increase vulnerability to parental burnout. The limited overlap found in the current study between professional and parental burnout suggests that they probably share common causes but also rely on specific ones. At the micro-level, common causes may be a personality that scores highly for neuroticism, leading to the risk of burnout in several demanding settings, in particular family and work; specific causes for parental burnout may be personal history and its resulting ideal parental self (which may be unrealistic). At the meso-level, a common cause may be lack of support; while specific causes may for example lie in the number of children to care for, young, disabled or ill children, low household income, or inadequate parenting practices. At the macro-level, common causes may be conflicting values between the self-sacrifice of work or parenting and time for oneself. Specific causes may be laws and advertising that increase pressure on parents.

Regarding the consequences, positioning burnout on a continuum between parental stress and depression (Iacovides et al., 2003; Hakanen et al., 2008) suggests that depression may be a frequent consequence at the micro-system level. Other likely consequences include the risk of addiction and deteriorating health, as previously demonstrated for job burnout (Ahola et al., 2006; Melamed et al., 2006). Consequences of these at the macro-system level would be a significant increase in health care costs. At the meso-level, parental disengagement and low accomplishment may lead to a reduction of responsiveness (Bornstein, 1989), which is known to be related to poor parent-child relationships and insecure attachment (van Ijzendoorn, 1995), harsh, neglecting parenting or maltreatment (Aber and Allen, 1987; Wiggins et al., 2015). Because of the potentially dramatic and long-lasting consequences that parental burnout may have for children, parental burnout's prospective effect on child development as well as behavioral issues should be a top priority in the research agenda. As well as the child, parental burnout certainly impacts the partner, who has to compensate for his/her coparent's withdrawal from family life and/or neglectful behavior toward offspring. A negative effect of parental burnout on conjugal conflict and coparenting is also expected. Finally, our experience with children suffering from externalized disorders suggests that parental burnout may also increase the risk of separation and divorce.

The foregoing research is necessary to build a comprehensive model of parental burnout, including both its underlying mechanisms and prospective effect at all levels. This model would offer insight into therapeutic solutions, the relevance and efficiency of which in reducing parental burnout should finally be tested with an evidence-based approach. Systematic comparisons of effect sizes resulting from interventions focusing on the macro-, meso- and micro-level mechanisms are needed. They should inform researchers and clinicians about the best ways to decrease the impact of risk factors or to increase the effect of protective ones, as well as to limit the harmful consequences for the parent him/herself, the coparent and their children. As this paper suggests, there are many interesting and socially relevant research directions in the field of parental burnout, as much as there were in the field of job burnout 40 years ago. We hope that the present contribution, in particular the development and validation of an instrument to measure parental burnout, will stimulate research in this area.

## Ethics statement

This study was carried out in accordance with the recommendations of APA Ethical Principles of Psychologists and Code of Conduct with written informed consent from all subjects. All subjects gave written informed consent in accordance with the Declaration of Helsinki. The protocol was approved by the Psychological Sciences Research Institute of the Université catholique de Louvain.

## Author contributions

IR designed the project, lead the data collection, computed the analyses, wrote the Method, Results, and Discussion sections. MR lead the data collection and computed preliminary analyses. MM designed the project, lead the data collection, wrote the Introduction and Discussion sections.

### Conflict of interest statement

The authors declare that the research was conducted in the absence of any commercial or financial relationships that could be construed as a potential conflict of interest.
