# Corrigendum: Exhausted Parents: Development and Preliminary Validation of the Parental Burnout Inventory

**DOI:** 10.3389/fpsyg.2018.00073

**Published:** 2018-01-30

**Authors:** Isabelle Roskam, Marie-Emilie Raes, Moïra Mikolajczak

**Affiliations:** Psychological Sciences Research Institute, Université Catholique de Louvain, Louvain-la-Neuve, Belgium

**Keywords:** parent, burnout, exhaustion, questionnaire, test, psychometrics

In the original article, there was a mistake in Table 4 as published. The copyright holders did not give the permission to include the items EE1 to EE8 and PA1 to PA6. Copyright of items was missing: Items EE1 to EE8 and PA1 to PA6 Copyright © 1981 Christina Maslach & Susan E. Jackson. All rights reserved in all media. Published by Mind Garden, Inc., www.mindgarden.com. Altered with permission of the publisher.

**Table 4:** Standardized regression weights from CFA and reliability estimates for the final 22-item version^1^ in study 2.

	**ED**	**EE**	**PA**
ED1	0.587		
ED2	0.461		
ED3	0.533		
ED4	0.749		
ED5	0.671		
ED6	0.558		
ED7	0.757		
ED8	0.489		
EE1		0.815	
EE2		0.721	
EE3		0.741	
EE4		0.850	
EE5		0.767	
EE6		0.837	
EE7		0.698	
EE8		0.903	
PA1			0.414
PA2			0.434
PA3			0.587
PA4			0.879
PA5			0.722
PA6			0.726
α	0.88	0.95	0.87

*PA, Personal Accomplishment; EE, Emotional Exhaustion; ED, Emotional Distancing*.

1 *Items EE1 to EE8 and PA1 to PA6 Copyright © 1981 Christina Maslach & Susan E. Jackson. All rights reserved in all media. Published by Mind Garden, Inc., www.mindgarden.com. Altered with permission of the publisher*.

In the Supplementary Material, there was a mistake in Table S1 as published. The copyright holders did not give the permission to include the items PA1 to DP5. Copyright of items was missing: Copyright © 1981 Christina Maslach & Susan E. Jackson. All rights reserved in all media. Published by Mind Garden, Inc., www.mindgarden.com. Altered with permission of the publisher.

In the Supplementary Material, there was a mistake in Table S2 as published. The copyright holders did not give the permission to include the items PA1 to PA8 and EE1 to EE9. Copyright of items was missing: Items PA1 to PA8 and EE1 to EE9 Copyright © 1981 Christina Maslach & Susan E. Jackson. All rights reserved in all media. Published by Mind Garden, Inc., www.mindgarden.com. Altered with permission of the publisher.

The authors apologize for these errors and state that this does not change the scientific conclusions of the article in any way.

The original article has been updated.

## Conflict of interest statement

The authors declare that the research was conducted in the absence of any commercial or financial relationships that could be construed as a potential conflict of interest.

