# Supplementary material for: Exhausted Parents: Development and Preliminary Validation of the Parental Burnout Inventory
Source: Front Psychol. 2017 Feb 9;8:163. doi: 10.3389/fpsyg.2017.00163 (PMC5298986; doi:10.3389/fpsyg.2017.00163)
Supplement: Supplementary file 1 [file Table1.DOCX]

*S1* Loading parameter estimates in PCA for the three-factor solution and reliability estimates in study 1^[[1]](#footnote-1)^

|  | PA | EE | DP |
| --- | --- | --- | --- |
| PA1 | **.718** | .237 | -.052 |
| PA2 | **.818** | .258 | -.004 |
| PA3 | **.804** | .144 | -.026 |
| PA4 | **.389** | -.166 | -.178 |
| PA5 | **.690** | -.149 | -.003 |
| PA6 | **.620** | -.216 | .084 |
| PA7 | **.710** | -.013 | .118 |
| PA8 | **.687** | .003 | -.056 |
| EE1 | .093 | **.840** | .043 |
| EE2 | .169 | **.831** | .086 |
| EE3 | .122 | **.822** | .135 |
| EE4 | -.038 | **.804** | .036 |
| EE5 | .063 | **.878** | .090 |
| EE6 | -.132 | **.716** | -.075 |
| EE7 | -.058 | **.759** | -.036 |
| EE8 | -.050 | **.813** | -.099 |
| EE9 | -.023 | **.828** | .010 |
| DP1 | -.121 | **.349** | .167 |
| DP2 | .001 | -.069 | **.849** |
| DP3 | -.057 | .006 | **.780** |
| DP4 | -.270 | -.089 | .108 |
| DP5 | -.050 | **.483** | -.249 |
|  | .89 | .95 | .81 |

*Note* Factor loadings >|.30| are in bold; PA: Personal Accomplishment; EE: Emotional Exhaustion; DP: Depersonalization.

1. Copyright © 1981 Christina Maslach & Susan E. Jackson.   All rights reserved in all media.  Published by Mind Garden, Inc., [www.mindgarden.com](http://www.mindgarden.com/). Altered with permission of the publisher. [↑](#footnote-ref-1)
