# Supplementary material for: Exhausted Parents: Development and Preliminary Validation of the Parental Burnout Inventory
Source: Front Psychol. 2017 Feb 9;8:163. doi: 10.3389/fpsyg.2017.00163 (PMC5298986; doi:10.3389/fpsyg.2017.00163)
Supplement: Supplementary file 2 [file Table2.DOCX]

*S2* Loading parameter estimates in PCA for the three-factor solution and reliability estimates for the 28-item version in study 2^[[1]](#footnote-1)^

|  | PA | EE | ED |
| --- | --- | --- | --- |
| PA1 | **-.680** | -.261 | .092 |
| PA2 | **-.703** | -.287 | .150 |
| PA3 | **-.697** | -.077 | .095 |
| PA4 | **-.693** | .288 | -.100 |
| PA5 | **-.648** | -.059 | .090 |
| PA6 | **-.740** | .300 | -.283 |
| PA7 | **.699** | .272 | -.069 |
| PA8 | **.716** | .094 | -.073 |
| ED1 | .003 | .054 | **.653** |
| ED2 | .022 | -.008 | **.604** |
| ED3 | .124 | -.055 | **.808** |
| ED4 | -.011 | .252 | **.574** |
| ED5 | -.016 | -.004 | **.809** |
| ED6 | -.106 | -.039 | **.685** |
| ED7 | .026 | .**375** | **.498** |
| ED8 | .034 | .155 | **.433** |
| ED9 | **.713** | -.270 | .115 |
| ED10 | **.669** | .075 | -.071 |
| ED11 | **.760** | -.286 | .296 |
| EE1 | .007 | **.874** | -.082 |
| EE2 | .089 | **.869** | -.106 |
| EE3 | .061 | **.796** | .008 |
| EE4 | -.037 | **.757** | .112 |
| EE5 | .016 | **.901** | -.016 |
| EE6 | -.055 | **.575** | .232 |
| EE7 | -.079 | **.668** | .197 |
| EE8 | -.049 | **.729** | .177 |
| EE9 | **.723** | .298 | -.129 |
|  | .93 | .94 | .89 |

*Note* Factor loadings >|.30| are in bold; PA: Personal Accomplishment; EE: Emotional Exhaustion; ED: Emotional Distancing.

1. Items PA1 to PA8 and EE1 to EE9 Copyright © 1981 Christina Maslach & Susan E. Jackson.   All rights reserved in all media.  Published by Mind Garden, Inc., [www.mindgarden.com](http://www.mindgarden.com/). Altered with permission of the publisher. [↑](#footnote-ref-1)
